# Sex and Exercise Intensity Do Not Influence Oxygen Uptake Kinetics in Submaximal Swimming

**DOI:** 10.3389/fphys.2017.00072

**Published:** 2017-02-10

**Authors:** Joana F. Reis, Gregoire P. Millet, Paula M. Bruno, Veronica Vleck, Francisco B. Alves

**Affiliations:** ^1^Laboratory of Physiology and Biochemistry of Exercise, Faculty of Human Kinetics, University of LisbonLisbon, Portugal; ^2^Ciper, Faculty of Human Kinetics, University of LisbonLisbon, Portugal; ^3^Universidade EuropeiaLisbon, Portugal; ^4^Department of Physiology, Faculty of Biology and Medicine, Institute of Sport Sciences, University of LausanneLausanne, Switzerland

**Keywords:** trained swimmers, time constant, slow component, oxygen consumption, female swimmers

## Abstract

The aim of this study was to compare the oxygen uptake ($\dot{\text{V}}{\text{O}}_{2}$) kinetics in front crawl between male and female swimmers at moderate and heavy intensity. We hypothesized that the time constant for the primary phase $\dot{\text{V}}{\text{O}}_{2}$ kinetics was faster in men than in women, for both intensities. Nineteen well trained swimmers (8 females mean ± SD; age 17.9 ± 3.5 years; mass 55.2 ± 3.6 kg; height 1.66 ± 0.05 m and 11 male 21.9 ± 2.8 years; 78.2 ± 11.1 kg; 1.81 ± 0.08 m) performed a discontinuous maximal incremental test and two 600-m square wave transitions for both moderate and heavy intensities to determine the $\dot{\text{V}}{\text{O}}_{2}$ kinetics parameters using mono- and bi-exponential models, respectively. All the tests involved breath-by-breath analysis of front crawl swimming using a swimming snorkel. The maximal oxygen uptake $\left(\dot{\text{V}}{\text{O}}_{2\text{max}}\right)$ was higher in men than in women [4,492 ± 585 ml·min^−1^ and 57.7 ± 4.4 ml·kg^−1^·min^−1^ vs. 2,752.4 ± 187.9 ml·min^−1^ (*p* ≤ 0.001) and 50.0 ± 5.7 ml·kg^−1^·min^−1^(*p* = 0.007), respectively]. Similarly, the absolute amplitude of the primary component was higher in men for both intensities (moderate: 1,736 ± 164 vs. 1,121 ± 149 ml·min^−1^; heavy: 2,948 ± 227 vs. 1,927 ± 243 ml·min^−1^, *p* ≤ 0.001, for males and females, respectively). However, the time constant of the primary component (τ_p_) was not influenced by sex (*p* = 0.527) or swimming intensity (*p* = 0.804) (moderate: 15.1 ± 5.6 vs. 14.4 ± 5.1 s; heavy: 13.5 ± 3.3 vs. 16.0 ± 4.5 s, for females and males, respectively). The slow component in the heavy domain was not significantly different between female and male swimmers (3.2 ± 2.4 vs. 3.8 ± 1.0 ml·kg^−1^·min^−1^, *p* = 0.476). Overall, only the absolute amplitude of the primary component was higher in men, while the other $\dot{\text{V}}{\text{O}}_{2}$ kinetics parameters were similar between female and male swimmers at both moderate and heavy intensities. The mechanisms underlying these similarities remain unclear.

## Introduction

In cyclic sports, such as swimming, running, or rowing, after the start of the race, the changes in metabolic rate are rather large and fast, forcing the cardiorespiratory system to respond promptly and precisely to prevent large variations of arterial blood gas and acid-base status (Burnley and Jones, 2007). The pulmonary oxygen uptake ($\dot{\text{V}}{\text{O}}_{2}$) kinetics is considered to be a useful, non-invasive measure of the integrated capacity of the organism to transport and utilize O_2_ in order to support the increased rate of muscular energy turnover. It provides an important assessment of the physiological response of the athlete (Jones and Carter, 2000; Burnley and Jones, 2007). When constant-load exercise is performed at moderate intensity, i.e., below the first ventilatory threshold (VT), after the cardiodynamic phase, the $\dot{\text{V}}{\text{O}}_{2}$ rises mono-exponentially until a steady state is achieved. In heavy intensity domain, i.e., above the VT, the attainment of a steady state in $\dot{\text{V}}{\text{O}}_{2}$ is delayed by a supplemental rise in $\dot{\text{V}}{\text{O}}_{2}$ or “slow component” (Barstow and Molé, 1991; Borrani et al., 2001; Carter et al., 2002). The time constant of the $\dot{\text{V}}{\text{O}}_{2}$ response (τ_p_; time for completion of 63% of the response) within the transition between two levels of energy requirement is typically around 20–35 s for young healthy subjects (Borrani et al., 2001; Carter et al., 2002; Poole and Jones, 2005).

Numerous studies have described the $\dot{\text{V}}{\text{O}}_{2}$ kinetics parameters within constant load cycling or running exercise (Whipp and Wasserman, 1972; Carter et al., 2000, 2002; Borrani et al., 2001; Murias et al., 2014), however, $\dot{\text{V}}{\text{O}}_{2}$ kinetics in swimming is considerably less studied (Rodriguez et al., 2003; Filho et al., 2012; Reis et al., 2012a,b; Sousa et al., 2015). In well-trained swimmers, the values reported for τ_p_ in heavy and severe intensities are similar to those reported in running and rowing (Reis et al., 2012a,b; Espada et al., 2014; Sousa et al., 2015). Similarly to other sports, faster $\dot{\text{V}}{\text{O}}_{2}$ kinetics in swimming have been associated with performance in middle distance events (Rodriguez et al., 2003; Reis et al., 2012b; Espada et al., 2014).

It has been shown that women have lower respiratory and cardiovascular capacities than their male counterpart. Namely, women have smaller stroke volumes, cardiac outputs, arterial oxygen content, hemoglobin and less red blood cells concentration than men in rest and in submaximal exercise both in absolute values or relative to body surface values (Wiebe et al., 1998; Wheatley et al., 2014). Furthermore, women also present smaller lung volumes, lower resting lung diffusion capacity and lower maximal expiratory flow rates (Harms, 2006). Therefore, it is not surprising that men present higher absolute $\dot{\text{V}}{\text{O}}_{2\text{max}}$ and produce higher power outputs than women (Wiebe et al., 1998). Although the lower cardiac and respiratory capacities of women in rest and exercise could induce smaller O_2_ delivery and utilization to the muscle, and consequently, slower $\dot{\text{V}}{\text{O}}_{2}$ kinetics than men, women also present an enhanced blood flow to the working muscle, namely they present higher blood flow and vascular conductance for the femoral blood flow in steady state exercise (Parker et al., 2007). Conversely, in the forearm exercise the vasodilatory responses do not differ between men and women (Limberg et al., 2010). Furthermore, it should be acknowledged that the microvascular O_2_ delivery could be a determinant factor in the $\dot{\text{V}}{\text{O}}_{2}$ kinetics response, which is not expected to be influenced by sex (Murias et al., 2014). Thus, although the current physiological models seem to support the similarity the response between men and women, there are still some sex induced differences that can potentially influence the oxygen uptake kinetics response. Furthermore, since in swimming the training groups frequently include both men and women, it is of most importance to verify the possible differences in order to increase the specificity of the training bouts.

The characterization of $\dot{\text{V}}{\text{O}}_{2}$ kinetics in females has been considerably less studied than in males and the literature has provided conflicting results regarding the differences between sexes (Fawkner and Armstrong, 2003; Murias et al., 2010, 2011a). While for heavy exercise prepubertal boys present faster $\dot{\text{V}}{\text{O}}_{2}$ kinetics and smaller contribution of the slow component than maturational age matched girls, there are no differences in $\dot{\text{V}}{\text{O}}_{2}$ kinetics between male and female children and adults in moderate exercise (Fawkner and Armstrong, 2003). Also in moderate exercise Murias et al. (2010, 2011a) reported similar $\dot{\text{V}}{\text{O}}_{2}$ Kinetics for men and women of similar fitness levels. Conversely, recently Lai et al. (2016) reported that adolescent women had slower $\dot{\text{V}}{\text{O}}_{2}$ and heart rate kinetics in moderate and heavy intensity cycling and presented a higher slow component for the latter.

In swimming, the literature has mainly presented data for male swimmers: to the best of our knowledge, only one study has characterized separately the $\dot{\text{V}}{\text{O}}_{2}$ kinetics for female swimmers (Rodriguez et al., 2003). However, these researchers only studied 4 female swimmers, describing the $\dot{\text{V}}{\text{O}}_{2}$ kinetics for race-pace velocities in 100 and 400 m front crawl swimming.

Since the control for $\dot{\text{V}}{\text{O}}_{2}$ kinetics across exercise domains can be attributed to different physiological mechanisms (Carter et al., 2002; Poole and Jones, 2005), it is important to study the influence of sex on $\dot{\text{V}}{\text{O}}_{2}$ kinetics both below and above the ventilatory threshold. Additionally, to date, most studies in swimming described the oxygen uptake kinetics above VT, i.e., for heavy, severe and extreme exercise (Sousa et al., 2011; Filho et al., 2012; Reis et al., 2012a,b). To the best of our knowledge only two studies analyzed the $\dot{\text{V}}{\text{O}}_{2}$ kinetics response in moderate swimming and used only one repetition (Sousa et al., 2013) and/or reported the values obtained in an incremental protocol (de Jesus et al., 2015). The comparison between τ_p_ for moderate and heavy exercise, using multiple exercise transitions has yet to be done, which can be useful to describe the control of on-kinetics in such exercise modality. Furthermore, in swimming most of the water-training is performed at these intensities and the volume performed at such intensities is significantly correlated with performance (Mujika et al., 1996).

Therefore, the aim of the present study was to compare the $\dot{\text{V}}{\text{O}}_{2}$ kinetics response of male and female trained swimmers during moderate and heavy intensity exercise. We hypothesized that the $\dot{\text{V}}{\text{O}}_{2}$ dynamic response is faster in males than in females in moderate and heavy intensity swimming.

## Materials and methods

### Subjects

Nineteen (8 females mean ± SD; age 17.9 ± 3.5 years; mass 55.2 ± 3.6 kg; height 1.66 ± 0.05 m; number of weekly training sessions 8.9 ± 0.6 and 11 male 21.9 ± 2.8 years; 78.2 ± 11.1 kg; 1.81 ± 0.08 m; number of weekly training sessions 8.7 ± 1.1) well-trained swimmers of national and international level participated in this study. All the subjects had been previously familiarized with the test procedures and equipment used in the experiment.

This study was carried out in accordance with the recommendations of Scientific Committee of the Faculty of Human Kinetics of the University of Lisbon with written informed consent from all subjects. All subjects gave written informed consent in accordance with the Declaration of Helsinki. The protocol was approved by the Scientific Committee of the Faculty of Human Kinetics of the University of Lisbon.

### Design

Oxygen uptake was measured during all test sessions using a breath-by-breath analyzer system (K4b2, Cosmed, Italy), calibrated immediately before each test according to the manufacturer's instructions. The analyzer was connected to the swimmer by a respiratory snorkel and valve system (Aquatrainer, Cosmed, Italy), previously validated for the determination of $\dot{\text{V}}{\text{O}}_{2}$ kinetics by our research group (Reis et al., 2010).

The tests were performed only in front crawl due to constraints of using the respiratory snorkel, with in-water starts and open turns and without underwater gliding. Target velocities were adjusted for each swimmer according to personal best times, and controlled on the basis of acoustic feedback to the swimmers in each 25 m.

All tests were conducted under the same conditions of environmental temperature, humidity and time of day and the subjects were instructed to report to the pool in a rested, fully hydrated state, at least 2 h after eating, having avoided strenuous exercise in the 24 h before a test session.

### Incremental test

The swimmers first performed an incremental test to exhaustion comprising 5 × 200 m sets with 30 s rest intervals, for determination of maximal oxygen uptake $\left(\dot{\text{V}}{\text{O}}_{2\text{max}}\right)$ and the first ventilatory threshold (VT) (Roels et al., 2005; Reis et al., 2012b). The velocity of the first repetition was calculated as 60% of the subject's best season competition time for 200 m, and 5–10% velocity increments between the first and fourth repetition were imposed. The last repetition was performed at maximal velocity $\left(\text{v}\dot{\text{V}}{\text{O}}_{2\text{max}}\right)$. $\dot{\text{V}}{\text{O}}_{2\text{max}}$ was designated as the highest 30 s $\dot{\text{V}}{\text{O}}_{2}$ average. VT was established as the oxygen uptake at which $\dot{\text{VE}}/\dot{\text{V}}{\text{O}}_{2}$ and end-tidal O_2_ pressure (PETO_2_) began to increase without a simultaneous increase in end-tidal CO_2_ pressure (PETCO_2_) (Wasserman et al., 1973). Heart rate (HR) was recorded telemetrically at 5 s intervals (Polar RS800, Kempele, Finland). Immediately after each repetition, fingertip blood lactate concentration was determined (Arkray, Kyoto, Japan). Lactate concentration [La] was also analyzed 3, 5, and 7 min after the end of exercise, for the determination of maximal lactate concentration (La_max_).

### Square-wave transitions

On subsequent days, the swimmers performed, in this order, two 600-m constant velocity swimming bouts corresponding to 80% VT (Moderate) and 25% Δ [VT + 0.25 x ($\dot{\text{V}}{\text{O}}_{2\text{max}}$ − VT)] (Heavy), respectively. The swimming bouts were separated by 10 min of passive rest. For all the subjects, said inter-bout rest times assured that the $\dot{\text{V}}{\text{O}}_{2}$ and [La] returned to rest values.

The above procedure was repeated by all the subjects within 1 week of its first completion. Thus, $\dot{\text{V}}{\text{O}}_{2}$ kinetics data were obtained for a total of two repetitions for each exercise transition. Throughout each swimming bout, the heart rate was measured continuously and immediately after, the [La] was determined, using the same procedure as in the incremental test.

### Data handling

For each transition, only the first 7 min of exercise were considered for the analysis.

The breath-by-breath values lying more than three standard deviations from the local mean were previously removed from the data. The data of the two square-wave transitions for moderate and heavy swimming were then interpolated into 1-s values, time-aligned, and ensemble averaged to provide a single on-transient set of data for each swimming transition.

$\dot{\text{V}}{\text{O}}_{2}$ kinetics parameters were calculated, by an iterative procedure, minimizing the sum of the residuals (squares of the differences between the modeled and the measured $\dot{\text{V}}{\text{O}}_{2}$ values), according to the following equation:

(1) $$\dot{V}O_{2}\left(t\right)=\{\begin{array}{lll} \dot{V}O_{2base} & \text{for }t<td_{p} & \;\\ \dot{V}O_{2base}+A_{p}\left(1-e^{-\left(t-td_{p}\right)\;/\tau_{p}}\right) & \text{for }td_{p}\leq t<td_{sc} & \left(\begin{array}{l} \text{primary}\\ \text{component} \end{array}\right)\\ \dot{V}O_{2base}+A_{p}\left(1-e^{-\left(td_{sc}-td_{p}\right)\;/\tau_{p}}\right)+A_{sc}\left(1-e^{-\left(t-td_{sc}\right)/\tau_{sc}}\right) & \text{for }t\geq td_{sc} & \left(\begin{array}{l} \text{slow}\\ \text{component} \end{array}\right) \end{array}$$

where $\dot{\text{V}}{\text{O}}_{2}\left(t\right)$ represents the relative $\dot{\text{V}}{\text{O}}_{2}$ at a given time, $\dot{V}O_{2base}$ represents the rest $\dot{\text{V}}{\text{O}}_{2}$ calculated as the average $\dot{\text{V}}{\text{O}}_{2}$ of the first 30 s of the last minute before exercise, td_p_, τ_p_, A_p_ represent the time delay, the time constant and the amplitude of the primary phase and slow component, and td_sc_, τ_sc_, A_sc_, represent the same parameters for the slow component.

Since Whipp et al. (1982) stated that some 20 s after the start of the exercise, the primary component begins, and based on an experimental approach designed by Murias et al. (2011b) we chose to exclude the first 20 s of data from the analysis to remove the influence of the cardiodynamic phase on the subsequent response.

For the moderate swimming exercise the slow component was not considered, since the monoexponential model was the best fit in all subjects.

For the heavy transitions, because the asymptotic value of the second function is not necessarily reached at the end of the exercise, the amplitude of the $\dot{\text{V}}{\text{O}}_{2}$ slow component was defined as $A_{sc}^{\prime }\;=\;A_{sc}\;(1-e^{-(te-td_{sc})/\tau_{sc}\;})$ where *te* was the time at the end of the exercise bout (Borrani et al., 2001).

The modeling for both intensities incorporated an individual “snorkel delay” (ISD) validated in previous work by our research group (Reis et al., 2010). ISD was calculated for each subject repetition as the difference between the onset of exercise (t_s_) and the time (t_ISD_) when the following breaths summed a tidal volume (TV) superior to the outlet tube volume (RSV), i.e., when the $\dot{\text{V}}{\text{O}}_{2}$ data so obtained could be considered to be representative of the exercise task.

The primary component (Gain A_p_) and end-exercise (EEgain) gain were computed by dividing the A_p_ or the End-Exercise $\dot{\text{V}}{\text{O}}_{2}$ (EE $\dot{\text{V}}{\text{O}}_{2}$), respectively, by the Δ velocity.

### Statistical analysis

All statistical analyses were performed using the Statistical Package for the Social Sciences (SPSS Statistics 20.0 for Windows, SPSS Inc., Chicago, USA). Normality of the distribution was checked by the Shapiro-Wilk's test. The data was then analyzed using a mixed “between-within” analysis of variance (ANOVA), with sex as a between-participant factor and exercise intensity as a within-participant factor. Statistical significance was accepted at *p* ≤ 0.05.

## Results

The swimmers responses obtained in the incremental test and in the square-wave transitions are given in Tables 1, 2, respectively. In the incremental test, male swimmers showed higher absolute and relative $\dot{\text{V}}{\text{O}}_{2\text{max}}$ and $\text{v}\dot{\text{V}}{\text{O}}_{2\text{max}}$ (*p* = 0.000). Conversely, male and female swimmers presented similar values of maximal heart rate and maximal lactate concentration, as well as VT expressed as a percentage of $\dot{\text{V}}{\text{O}}_{2\text{max}}$.

**Table 1:** **Mean and standard deviation (SD) of the aerobic parameters obtained in the incremental test for men and women**.

**Variable**	**Men**	**Women**
$\dot{\text{V}}{\text{O}}_{2\text{max}}$ (ml kg^−1^ min^−1^)	57.7 ± 4.4	50.0 ± 5.7^a^
$\dot{\text{V}}{\text{O}}_{2\text{max}}$ (ml min^−1^)	4492.5 ± 585.5	2752.4 ± 187.9^a^
$\text{v}\dot{\text{V}}{\text{O}}_{2\text{max}}$ (m s^−1^)	1.49 ± 0.06	1.33 ± 0.05^a^
VT (% $\dot{\text{V}}{\text{O}}_{2\text{max}}$)	75.8 ± 6.8	77.9 ± 5.5
La_max_ (mmol l^−1^)	10.3 ± 2.2	8.1 ± 2.5
HR_max_ (beats min^−1^)	181.9 ± 7.6	193.8 ± 9.7

*$\dot{V}O_{\mathrm{2max}}$, relative and absolute maximal oxygen consumption; $v\dot{V}O_{\mathrm{2max}}$, velocity associated to $\dot{V}O_{\mathrm{2max}}$; VT, oxygen consumption at the ventilatory threshold relative to $\dot{V}O_{\mathrm{2max}}$; La_max_, maximal lactate concentration; HR_max_, maximal heart rate*.

a *Significantly different than men for the same intensity (p < 0.05)*.

**Table 2:** **Mean ± SD parameters of the ${\dot{\text{V}}\text{O}}_{2}$ kinetics for transition from rest to 80% VT (moderate) and Δ25% (heavy) for Men and Women**.

**Variables**	**Moderate**		**Heavy**	
	**Men**	**Women**	**Men**	**Women**
$\dot{V}O_{2}base$ (ml kg^−1^ min^−1^)	8.86 ± 1.2	8.07 ± 1.1	8.1 ± 1.7	7.8 ± 1.4
$\dot{V}O_{2}base$ (ml min^−1^)	692 ± 132	445 ± 60^a^	633 ± 132	430 ± 77^a^
A_p_ (ml kg^−1^ min^−1^)	22.2 ± 2.1	20.3 ± 2.7^a^	37.7 ± 2.9^b^	34.9 ± 4.4^b^
A_p_ (ml min^−1^)	1736 ± 164	1121 ± 149^a^	2948 ± 227^b^	1927 ± 243^a^^,^^b^
td_p_ (s)	12.3 ± 4.3	12.7 ± 2.8	11.1 ± 3.7	11.2 ± 4.7
τ_p_ (s)	14.4 ± 5.1	15.1 ± 5.6	16.0 ± 4.5	13.5 ± 3.3
A_sc_' (ml kg^−1^ min^−1^)			3.8 ± 1.0	3.2 ± 2.4
A_sc_' (ml min^−1^)			297 ± 78	177 ± 132
%A_sc_'			7.5 ± 1.8	6.6 ± 3.8
tdsc (s)			169.1 ± 70.0	167.5 ± 51.9
τ_sc_ (s)			92.0 ± 123.7	36.3 ± 36.2
Gain Ap (ml·min^−1^·m^−1^)	13.9 ± 7.6	11.4 ± 3.2	28.7 ± 4.7^a^	20.5 ± 3.4^a^^,^^b^
EE Gain (ml·min^−1^·m^−1^)	27.5 ± 3.9	18.6 ± 4.0^a^	40.5 ± 4.7^b^	28.6 ± 4.5^a^^,^^b^
EE $\dot{\text{V}}{\text{O}}_{2}$ (ml kg^−1^ min^−1^)	31.5 ± 2.6	28.1 ± 3.5^a^	49.5 ± 3.1^b^	45.7 ± 6.5^b^
EE $\dot{\text{V}}{\text{O}}_{2}$ (% VO_2max_)	54.9 ± 6.1	56.6 ± 6.5	86.2 ± 7.7^b^	91.6 ± 9.6^b^
EE HR (b.min^−1^)	125.2 ± 7.3	130.7 ± 10.1	162.9 ± 8.3^b^	170.5 ± 10.9^b^
EE [La] (mmol l^−1^)	1.6 ± 0.6	1.5 ± 0.4	4.9 ± 1.7^b^	4.4 ± 1.7^b^
v (m s^−1^)	1.07 ± 0.07	1.00 ± 0.03	1.31 ± 0.07	1.21 ± 0.05

*$\dot{V}O_{2}$_base_, baselineVO_2_; Amplitude (A_p_, A_sc_), time delay (tdp, tdsc), time constant (τp, τsc), of the primary phase and slow component, respectively. %Asc': relative contribution of slow component in relation to the end-exercise $\dot{V}O_{2}$: EE $\dot{V}O_{2}$ − $\dot{V}O_{2}$ at the end of at the end of the constant load exercise; EE $\dot{V}O_{2}$ (% VO_2max_) − $\dot{\text{V}}{\text{O}}_{2}$ at the end of at the end of the constant load exercise relative to VO_2max_; EE HR: heart rate at the end of the constant load exercise; EE [La]: [La] at the end of exercise; v: swim velocity for each exercise intensity*.

a *Significantly different than men for the same intensity (p < 0.05)*,

b *Significantly different from moderate intensity swimming (p < 0.05)*.

**Figure 1:**
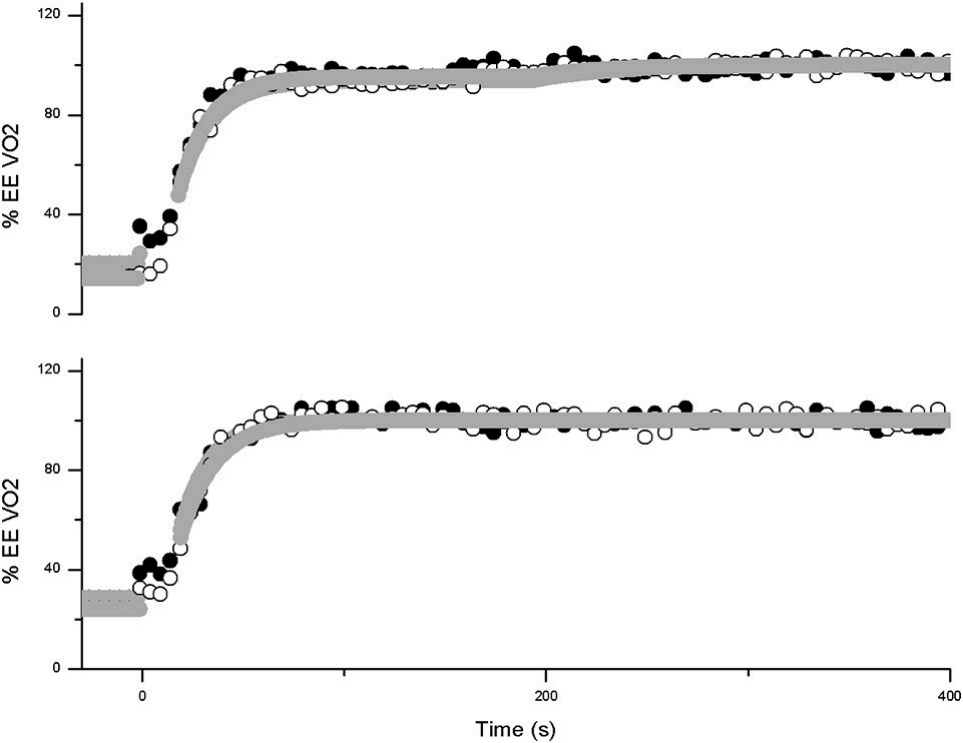
**$\dot{\text{V}}{\text{O}}_{2}$ response profile in two example subjects in a square wave transition to moderate (lower panel) and heavy (upper panel) swimming**. Breath-by-breath data of the female swimmer is shown in closed circles and of the male swimmer in open circles. The Gray lines represent the best fit as determined from the exponential modeling procedure (dark gray for the female and light gray for the male). The data is expressed as a percentage of the overall response.

**Figure 2:**
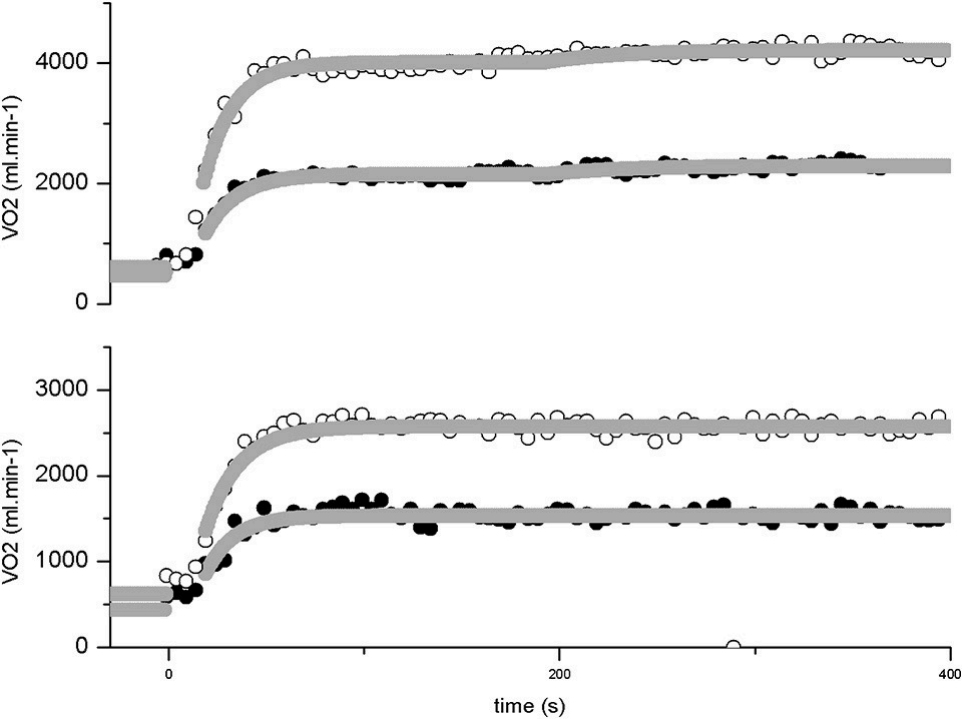
**${\dot{\text{V}}\text{O}}_{2}$ response profile in two example subjects in a square wave transition to moderate (lower panel) and heavy (upper panel) swimming**. Breath-by-breath data of the female swimmer is shown in closed circles and of the male swimmer in open circles. The Gray lines represent the best fit as determined from the exponential modeling procedure (dark gray for the female and light gray for the male). The data is expressed in absolute values.

$\dot{\text{V}}{\text{O}}_{2}$ responses for representative subjects in square wave transitions to Moderate and Heavy swimming are presented in Figures 1, 2. There was a significant effect of increased intensity in A_p_, EE $\dot{\text{V}}{\text{O}}_{2}$, EE HR and EE La, for both sexes (*p* = 0.000 for all parameters). For moderate exercise, the female swimmers presented lower relative values than men in A_p_ (*p* = 0.03) and EE $\dot{\text{V}}{\text{O}}_{2}$ (*p* = 0.04). The Gain A_p_ (*p* = 0.01) and EE gain for heavy exercise (*p* = 0.000) and EE gain for moderate exercise (*p* = 0.00) was significantly lower for females. However, τ_p_ and td_p_ were not influenced by either intensity (*p* = 0.804 and 0.326) nor sex (*p* = 0.527 and 0.908). Additionally, the *A*_*sc*_' was not different between men and women (*p* = 0.476).

## Discussion

The main finding of this work is that the time constant for the primary component of the $\dot{\text{V}}{\text{O}}_{2}$ kinetics response is similar between female and male trained swimmers in square transitions for both moderate and heavy intensity swimming.

Up to now, only one study has reported the $\dot{\text{V}}{\text{O}}_{2}$ kinetics separately for female swimmers (Rodriguez et al., 2003). Even so, the sample size was just 4 swimmers, only one transition per intensity was performed and it was limited to transitions for maximal intensity in 100 and 400 m race pace efforts. Therefore, the novelty of this study relies on the description of the $\dot{\text{V}}{\text{O}}_{2}$ kinetics in moderate and heavy intensity in highly trained swimmers of both sexes.

When absolute values were considered, male swimmers presented higher Ap at moderate and heavy intensities. However, we observed that only, A_p_ at moderate intensity was smaller in women. This difference is not surprising, since it has been shown that women have a lower energy cost than men at low intensity (Pendergast et al., 1977), which the authors credited to the differences in body size and velocity, both inducing higher values of drag for men. Furthermore, we also found a decreased gain, both for the primary component (in heavy swimming) and end-exercise in females. Since the gain reflects the energy consumption corrected for the distance, this fact could also be associated with the higher swimming economy of women.

Contrary to our hypothesis, we did not find differences in the primary phase time constant between sexes in either moderate or heavy swimming. It has been reported that women have smaller hearts, smaller stroke volumes, cardiac outputs and hemoglobin concentration than men (Wiebe et al., 1998). Furthermore, they have smaller lung volumes and lower maximal expiratory flow rates (Harms, 2006). This author suggested that during work above 80% of VO_2_max women are more susceptible to fatigue and exercise induced arterial hypoxemia. However, Murias et al. (2013) in a recent study reported that, in a maximal ramp test in the cycle ergometer, women presented higher O_2_ extraction for the same relative intensity than men, presenting a less effective matching of the O_2_ delivery and O_2_ utilization, which seem to translate impairments in blood flow. However, said lower oxygen delivery to the muscle and the cardiac and respiratory characteristics of women does not seem to affect the oxygen uptake kinetics in moderate and heavy swimming. Nevertheless, since men present higher absolute amplitudes for the primary component with similar time constants, the gross rate of increase of oxygen uptake per second is higher in men, suggesting a quicker onset. This could be due to the higher maximal oxygen uptake and larger muscle mass presented by the male swimmers.

Whereas the comparison between male and females remains to be thoroughly addressed in the literature for other exercise modalities, our results are in accordance to what was reported for middle age subjects in cycle ergometer, for heavy and moderate exercise (DeLorey et al., 2005; Connor et al., 2012) and for children and adults in moderate exercise (Fawkner and Armstrong, 2003). One of the reasons that can explain the fact that τ_p_ for transitions both below and above VT are similar between women and men of similar training backgrounds is that the intensity was not high enough for the oxygen delivery to the active muscle to be sufficiently compromised. In addition, since all the swimmers were highly trained and of similar performance level, the time constant of the oxygen uptake kinetics was likely already minimized (Murias et al., 2014) and, therefore, differences between male and female swimmers within our relatively homogeneous group of study participants were not detected.

Our results confirm the existence of a slow component, only for swimming intensities above the VT, similarly to what previous literature has reported for other sports (Carter et al., 2000, 2002). There were no differences in the slow component between male and females, both relative to body weight and to the end-exercise $\dot{\text{V}}{\text{O}}_{2}$ kinetics.

The values observed for τ_p_ for heavy intensity swimming are similar or somewhat faster than those reported from previous studies from the literature (Filho et al., 2012; Reis et al., 2012a,b; Espada et al., 2014). Namely, the average of the time constant for all our subjects was 14.9 ± 4.2 s, whereas previous studies reported τ_p_ of 17.3 ± 5.4 s, 15.8 ± 4.8 s and 16.9 ± 3.9 s for the same exercise intensity (Bentley et al., 2005; Reis et al., 2012a,b). However, these studies only evaluated male swimmers, whom in our study presented a τ_p_ of 16.0 ± 4.5 s for heavy intensity swimming. For higher intensities, namely race pace or severe swimming, a wider range of τ_p_ has been reported: from 10.5 ± 2.5 s for a 200 m race pace swimming bout in elite athletes (Sousa et al., 2011) to 21 ± 3 s for the square-wave transition to $\text{v}\dot{\text{V}}{\text{O}}_{2\text{max}}$ (Sousa et al., 2015). Due to the supine position and predominance of upper body use we could expect slower $\dot{\text{V}}{\text{O}}_{2}$ kinetics in swimming, however, surprisingly, most of the studies conducted in swimming, including the present, reported a kinetics similar to the upright exercise modalities (Sousa et al., 2011, 2015; Filho et al., 2012; Espada et al., 2014). One may speculate that the specific training adaptation surpass the possible impairments caused by the body position and muscle mass involved in swimming.

No differences were found in the τ_p_ between moderate and heavy intensity swimming in trained swimmers. Our work is in agreement with previous studies conducted in cycling and running that reported similar τ_p_ across exercise intensities (Barstow and Molé, 1991; Carter et al., 2002). According to Poole et al. (Poole and Jones, 2005) said invariant time constant across exercise intensities is generally attributed to the fact that the $\dot{\text{V}}{\text{O}}_{2}$ kinetics is restricted by metabolic inertia rather than oxygen delivery, since the oxygen delivery is compromised without similar decrease in the adaptation of the aerobic response. Therefore, since the time constant for heavy swimming is not slowed when the oxygen requirements of the exercise increases above the VT, one may suggest that in trained swimmers, with fast oxidative responses, the metabolic inertia, and not oxygen delivery, restricts $\dot{\text{V}}{\text{O}}_{2}$ kinetics at both moderate and heavy intensities. However, due to methodological constrains that restrict the measurements of muscle oxygenation and blood flow in swimming, the invariance of time constant cannot be categorically attributed to metabolic inertia, since for heavy intensity swimming the oxygen availability could not be sufficiently compromised to affect the immediate response of the aerobic system. Furthermore, the constrained breathing pattern imposed in front crawl swimming could potentially influence the O_2_ delivery when comparing swimming with terrestrial activities. We also must acknowledge the recent work of Murias et al. (2014) that suggested that in subjects with fast $\dot{\text{V}}{\text{O}}_{2}$ kinetics, such as the swimmers participants in these study, the intracellular control mechanisms are mainly responsible for the rate of adjustment of oxidative phosphorylation, representing an improved matching of O_2_ delivery (or distribution) to O_2_ utilization, a parameter that is was not possible to determine in our study. Therefore, the invariant time constant between exercise domains could also be a consequence of an improved vascular responsiveness and vascularization of the muscle caused by years of swimming training in these intensities.

This study also shows an unexpected increase in both the gain for the primary component and end exercise gain, between moderate and heavy swimming. This relative increase in energy cost could be associated with the increase in drag, which, contrary to other exercise modalities, induces a cubic relationship between energy expenditure and velocity (Pendergast et al., 1977). Of importance is that, from moderate to heavy domain, the increase in velocity and then in drag was similar between men and women.

## Practical applications

The exercise intensities used in our study are common in swimming training (Mujika et al., 1996). One of the practical applications of these findings for training is that coaches can prescribe interval-training similarly for both male and female swimmers of identical training background, since the most important $\dot{\text{V}}{\text{O}}_{2}$ kinetics parameters (i.e., time constant of the primary component and amplitude of the slow component) do not appear to be different for the same relative intensity. However, since there is a large interindividual variation in the $\dot{\text{V}}{\text{O}}_{2}$ response to swim interval training (Bentley et al., 2005), it is important for the coaches to have an individual evaluation of each swimmer, especially at the elite level.

## Limitations

Despite the encouraging results, some limitations are presented and should be considered: Although we acknowledge that the small number of subjects underpowered the present study, one may observe that the present sample size is within the usual range for similar studies with highly trained athletes.

We did not control the female swimmers for the menstrual cycle phase. However, Gurd et al. (2007) reported that there were no differences in either $\dot{\text{V}}{\text{O}}_{2}$ nor muscle deoxygenation kinetics between the follicular and luteal phases of the menstrual cycle in active women.

Future research should be conducted to refine this limitations.

## Conclusions

This study was the first to compare the $\dot{\text{V}}{\text{O}}_{2}$ kinetics between male and female trained swimmers in moderate and heavy intensity swimming, which are the most frequent intensities prescribed during training by swimming coaches. In highly trained individuals, the time constant of the primary component was not significantly different between sexes in both intensity domains and was also independent from swimming intensity. However, due to the instrumental limitations imposed by swimming exercise, namely, the inability to use oximetry in the water, we could not verify the physiological mechanisms responsible for this results.

## Author contributions

Conceived and designed the experiments: JR, GM, and FA. Performed experiments: JR. Analyzed data: JR, VV, and PB. Interpreted results of research: JR, GM, VV, and FA. Drafted manuscript and prepared tables/figures: JR and PB. Edited, critically revised paper and approved final version of manuscript: JR, GM, PB, VV, and FA.

## Funding

The first author gratefully acknowledges the “Fundação para a Ciência e Tecnologia, Portugal” (“The Foundation for Science and Technology, Portugal”) for their post-doctoral fellowship award (reference number SFRH/BPD/84315/2012). The results of the present study do not constitute endorsement of the mentioned instruments by the authors or the journal.

### Conflict of interest statement

The authors declare that the research was conducted in the absence of any commercial or financial relationships that could be construed as a potential conflict of interest.

## References

[B1] Barstow T. J. Molé P. A. (1991). Linear and nonlinear characteristics of oxygen uptake kinetics during heavy exercise. J. Appl. Physiol. 71, 2099–2106. 1778898 10.1152/jappl.1991.71.6.2099

[B2] Bentley D. J. Roels B. Hellard P. Fauquet C. Libicz S. Millet G. P. (2005). Physiological responses during submaximal interval swimming training: effects of interval duration. J. Sci. Med. Sport 8, 392–402. 10.1016/S1440-2440(05)80054-4 16602167

[B3] Borrani F. Candau R. Millet G. Y. Perrey S. Fuchslocher J. Rouillon J. D. (2001). Is the VO_2_ slow component dependent on progressive recruitment of fast-twitch fibers in trained runners? J. Appl. Physiol. 90, 2212–2220. 11356785 10.1152/jappl.2001.90.6.2212

[B4] Burnley M. Jones A. M. (2007). Oxygen uptake kinetics as a determinant of sports performance. Eur. J. Sport Sci. 7, 63–79. 10.1080/17461390701456148

[B5] Carter H. Jones A. M. Barstow T. J. Burnley M. Williams C. A. Doust J. H. (2000). Oxygen uptake kinetics in treadmill running and cycle ergometry: a comparison. J. Appl. Physiol. 89, 899–907. 10956332 10.1152/jappl.2000.89.3.899

[B6] Carter H. Pringle J. S. Jones A. M. Doust J. H. (2002). Oxygen uptake kinetics during treadmill running across exercise intensity domains. Eur. J. Appl. Physiol. 86, 347–354. 10.1007/s00421-001-0556-2 11990749

[B7] Connor E. O. Kiely C. Shea D. O. Green S. Egaña M. (2012). Similar level of impairment in exercise performance and oxygen uptake kinetics in middle-aged men and women with type 2 diabetes. Am. J. Physiol. Regul. Integr. Comp. Physiol. 303, 70–76. 10.1152/ajpregu.00012.2012 22538515

[B8] de Jesus K. Sousa A. de Jesus K. Ribeiro J. Machado L. Rodriguez F.. (2015). The effects of intensity on VO_2_ kinetics during incremental free swimming. Appl. Physiol. Nutr. Metab. 40, 918–923. 10.1139/apnm-2015-0029 26300011

[B9] DeLorey D. S. Kowalchuk J. M. Paterson D. H. (2005). Adaptation of pulmonary O_2_ uptake kinetics and muscle deoxygenation at the onset of heavy-intensity exercise in young and older adults. J. Appl. Physiol. 98, 1697–1704. 10.1152/japplphysiol.00607.2004 15640394

[B10] Espada M. C. Reis J. F. Almeida T. F. Bruno P. M. Vleck V. Alves F. B. (2014). Ventilatory and physiological responses in swimmers below and above their maximal lactate steady state. J. Strength Cond. Res. 29, 2836–2843. 10.1519/JSC.0000000000000504 25148466

[B11] Fawkner S. Armstrong N. (2003). Oxygen uptake kinetic response to exercise in children. Sports Med. 33, 651–669. 10.2165/00007256-200333090-00002 12846589

[B12] Filho D. M. P. Alves F. B. Reis J. F. Greco C. C. Denadai B. S.. (2012). VO_2_ kinetics during heavy and severe exercise in swimming. Int. J. Sports Med. 33, 744–748. 10.1055/s-0031-1299753 22592546

[B13] Gurd B. J. Scheid J. Paterson D. H. Kowalchuk J. M. (2007). O_2_ uptake and muscle deoxygenation kinetics during the transition to moderate-intensity exercise in different phases of the menstrual cycle in young adult females. Eur. J. Appl. Physiol. 101, 321–330. 10.1007/s00421-007-0505-9 17618450

[B14] Harms C. A. (2006). Does gender affect pulmonary function and exercise capacity? Respir. Physiol. Neurobiol. 151, 124–131. 10.1016/j.resp.2005.10.010 16406740

[B15] Jones A. M. Carter H. (2000). The effect of endurance training on parameters of aerobic fitness. Sport. Med. 29, 373–386. 10.2165/00007256-200029060-00001 10870864

[B16] Lai N. Martis A. Belfiori A. Tolentino-Silva F. Nasca M. M. Strainic J.. (2016). Gender differences in $\dot{\text{V}}$O_2_ and HR kinetics at the onset of moderate and heavy exercise intensity in adolescents. Physiol. Rep. 4, 1–12. 10.14814/phy2.12970 27655810 PMC5037918

[B17] Limberg J. K. Eldridge M. W. Proctor L. T. Sebranek J. J. Schrage W. G. (2010). Alpha-adrenergic control of blood flow during exercise: effect of sex and menstrual phase. J. Appl. Physiol. 109, 1360–1368. 10.1152/japplphysiol.00518.2010 20724565 PMC2980375

[B18] Mujika I. Chatard J. Busso T. Geyssant A. Barale F. Lacoste L. (1996). Use of swim-training profiles and performance data to enhance training effectiveness. J Swim. Res. 11, 23–29.

[B19] Murias J. M. Keir D. A. Spencer M. D. Paterson D. H. (2013). Sex-related differences in muscle deoxygenation during ramp incremental exercise. Respir. Physiol. Neurobiol. 189, 530–536. 10.1016/j.resp.2013.08.011 23994824

[B20] Murias J. M. Kowalchuk J. M. Paterson D. H. (2010). Speeding of VO_2_ kinetics with endurance training in old and young men is associated with improved matching of local O_2_ delivery to muscle O2 utilization. J. Appl. Physiol. 108, 913–922. 10.1152/japplphysiol.01355.2009 20150562 PMC2853203

[B21] Murias J. M. Kowalchuk J. M. Paterson D. H. (2011a). Speeding of VO_2_ kinetics in response to endurance-training in older and young women. Eur. J. Appl. Physiol. 111, 235–243. 10.1007/s00421-010-1649-6 20857137

[B22] Murias J. M. Spencer M. D. Kowalchuk J. M. Paterson D. H. (2011b). Influence of phase I duration on phase II VO_2_ kinetics parameter estimates in older and young adults. Am. J. Physiol. Regul. Integr. Comp. Physiol. 301, 218–224. 10.1152/ajpregu.00060.2011 21490368

[B23] Murias J. M. Spencer M. D. Paterson D. H. (2014). The critical role of O_2_ provision in the dynamic adjustment of oxidative phosphorylation. Exerc. Sport Sci. Rev. 42, 4–11. 10.1249/JES.0000000000000005 24188979

[B24] Parker B. A. Smithmyer S. L. Pelberg J. A. Mishkin A. D. Herr M. D. Proctor D. N.. (2007). Sex differences in leg vasodilation during graded knee extensor exercise in young adults. J. Appl. Physiol. 103, 1583–1591. 10.1152/japplphysiol.00662.2007 17717115

[B25] Pendergast D. R. Di Prampero P. E. Craig A. B. Wilson D. R. Rennie D. W. (1977). Quantitative analysis of the front crawl in men and women. J. Appl. Physiol. 43, 475–479. 914719 10.1152/jappl.1977.43.3.475

[B26] Poole D. Jones A. M. (2005). Understanding the mechanistic bases of VO_2_ kinetics. kinetics in sport, in Oxygen Uptake Kinetics in Sport, eds Poole D. Jones A. M. (London: Routledge), 294–328.

[B27] Reis J. F. Alves F. B. Bruno P. M. Vleck V. Millet G. P. (2012a). Effects of aerobic fitness on oxygen uptake kinetics in heavy intensity swimming. Eur. J. Appl. Physiol. 59, 1104–1109. 10.1007/s00421-011-2126-6 21879352

[B28] Reis J. F. Alves F. B. Bruno P. M. Vleck V. Millet G. P. (2012b). Oxygen uptake kinetics and middle distance swimming performance. J. Sci. Med. Sport 15, 58–63. 10.1016/j.jsams.2011.05.012 21802360

[B29] Reis J. F. Millet G. P. Malatesta D. Roels B. Borrani F. Vleck V. E.. (2010). Are oxygen uptake kinetics modified when using a respiratory snorkel? Int. J. Sports Physiol. Perform. 5, 292–300. 10.1123/ijspp.5.3.292 20861520

[B30] Rodriguez F. Keskinen K. Malvela M. Keskinen O. (2003). Oxygen uptake kinetics during free swimming: a pilot study, in Biomechanics and Medicine in Swimming IX, ed Chatard J. (Saint-Étienne: Publications de l'Université de Saint-Étienne), 379–384.

[B31] Roels B. Schmitt L. Libicz S. Bentley D. Richalet J.-P. Millet G. (2005). Specificity of VO_2max_ and the ventilatory threshold in free swimming and cycle ergometry: comparison between triathletes and swimmers. Br. J. Sports Med. 39, 965–968. 10.1136/bjsm.2005.020404 16306508 PMC1725090

[B32] Sousa A. de Jesus K. Figueiredo P. Vilas-Boas J. P. Fernandes R. J. (2013). Oxygen uptake kinetics at moderate and extreme swimming intensities. Rev. Bras. Med. do Esporte 19, 186–190. 10.1590/S1517-86922013000300008

[B33] Sousa A. Figueiredo P. Oliveira N. Oliveira J. Silva A. Keskinen K.. (2011). VO_2_ kinetics in 200-m race-pace front crawl. Int. J. Sport. Med. 32, 765–770. 10.1055/s-0031-1279772 21913155

[B34] Sousa A. Figueiredo P. Zamparo P. Pyne D. B. Vilas-Boas J. P. Fernandes R. J. (2015). Exercise modality effect on bioenergetical performance at VO_2max_ intensity. Med. Sci. Sports Exerc. 47, 1705–1713. 10.1249/MSS.0000000000000580 25412298

[B35] Wasserman K. Whipp B. J. Koyal S. N. Beaver W. L. (1973). Anaerobic threshold and respiratory gas exchange during exercise. J. Appl. Physiol. 35, 236–243. 4723033 10.1152/jappl.1973.35.2.236

[B36] Wheatley C. M. Snyder E. M. Johnson B. D. Olson T. P. (2014). Sex differences in cardiovascular function during submaximal exercise in humans. Springerplus 3:445. 10.1186/2193-1801-3-445 25191635 PMC4153874

[B37] Whipp B. J. Ward S. A. Lamarra N. Davis J. A. Wasserman K. (1982). Parameters of ventilatory and gas exchange dynamics during exercise. J. Appl. Physiol. 52, 1506–1513. 6809716 10.1152/jappl.1982.52.6.1506

[B38] Whipp B. J. Wasserman K. (1972). Oxygen uptake kinetics for various intensities of constant-load work. J. Appl. Physiol. 33, 351–356. 5056210 10.1152/jappl.1972.33.3.351

[B39] Wiebe C. G. Gledhill N. Warburton D. E. Jamnik V. K. Ferguson S. (1998). Exercise cardiac function in endurance-trained males versus females. Clin. J. Sport Med. 8, 272–279. 10.1097/00042752-199810000-00004 9884791

